# Cost-Effectiveness of Multifactorial Interventions in Preventing Falls among Elderly Population: A Systematic Review

**DOI:** 10.30476/BEAT.2021.84375.1068

**Published:** 2021-10

**Authors:** Vahid Alipour, Saber Azami-Aghdash, Aziz Rezapour, Naser Derakhshani, Akbar Ghiasi, Neghar Yusefzadeh, Sanaz Taghizade, Sahar Amuzadeh

**Affiliations:** 1 *Health Management and Economics Research Center, Iran University of Medical Sciences, Tehran, Iran*; 2 *Tabriz Health Services Management Research Center, Health Management and Safety Promotion Research Institute, Tabriz University of Medical Sciences, Tabriz, Iran *; 3 *Health Administration HEB School of Business & Administration, University of the Incarnate Word, Texas, USA *; 4 *Department of Health Management and Economics, School of Public Health, Tehran University of Medical Sciences, Tehran, Iran *; 5 *Babol University of Medical Sciences, Babol, Iran*

**Keywords:** Elderly, Falls, Cost-effectiveness, Intervention, Population

## Abstract

**Objective:**

To review the cost-effectiveness of multifactorial interventions to prevent falls in elderly people.

**Methods:**

In this systematic review, the databases including PubMed via MEDLINE, Web of Science, Embase, Scopus, Cochrane Library and Google Scholar (from 1st January 2000 to 30^th^ February) were used. All pre-reviewed articles related to cost-effectiveness analysis of multifactorial interventions to prevent falls in elderly were included in this paper and congresses abstracts were excluded. Descriptive statistics were used for quantitative data and content-analysis method to analyze qualitative data.

**Results:**

Out of the 456 articles, 19 were finally included in the study. Eighteen articles were conducted in High-Income Countries (HICs) and 16 were at the community level. Medical visits consultation and education were the most common interventions. Most studies were cost-effectiveness and using the Randomized Control Trial (RCT) methods. A fall of prevention costs ranged from $ 272 to $ 987. Incremental Cost-Effectiveness Ratio (ICER) interventions also ranged from the US $ 120,667 to the US $ 4280.9.

**Conclusion:**

The results show that despite the high effectiveness of multifactorial interventions to prevent elderly falls, the cost of the interventions are high and they are not very cost-effective. It would be better to design and implement multifactorial interventions with low cost and high effectiveness that are appropriate for each country.

## Introduction

Nowadays, all developed and underdeveloped countries are experiencing aging population worldly [1]. The world’s elderly population is estimated to surpass 1.9 billion by 2050 [2, 3]. Elderly population of the United States increased 15% above 65 years and 29.6% above 85 years old between 2000-2010 [4-7]. 

As the age of population increases, the health problems also increases [8]. In older ages, individuals are susceptible to more injuries, which leads to higher demand for healthcare services [9]. The elderly population compared with other age groups suffer worse conditions due to the physical conditions when traumatic injuries occur [10-15]. Moreover, the social costs such as loss of life are higher among this population due to trauma [16].

Currently, trauma is the 5^th^ leading cause of death among elderly with fall as the most common cause of trauma-related death [17, 18]. The more rates fall and its injuries are higher than the health care costs [19-21]. About One-third of people over 65 years and 50% of people over 85 years experiencing to fall each year [22]. The falls probability is estimated about 35% to 40% over a year in the elderly, which half of them can be more than a fall per person [23]. Falls and injuries are a widespread problem among the elderly population [24] with immediate and long-term consequences. According to studies, elderly fall accounts for About 80% of accident hospital admissions [25, 26] and 11% of the elderly death [27]. A study in Australia estimated the rate of falls in hospitalization of elderly people that is 30,000 in a year with 1% total death. The researchers showed high burden of diseases on the country’s healthcare system [28, 29]. “Loss of balance” and “loss of functional mobility” are two main reasons of fall in elderly population [30]. The other reasons of falls in the elderly are including of low ambient light, unsafe stairs, slippery rugs, inappropriate shoes, concurrent use of multiple drugs, psychedelic drugs use, musculoskeletal weakness, balance and gait disorder, visual impairment, nutritional disorder such as calcium and vitamin D deficiency and cardiovascular disease [31-33]. 

It is estimated that around 7% (€ 1.6 billion) of the total annual spending in National Health Systems (NHS) in UK was spent to treat traumatic injuries in 2008 by given the high cost of treating trauma injuries [34]. The United States has also allocated more than $ 35 billion of health care costs for the elderly between 2008 and 2013 [6]. 

Falls are influenced by several facilitating and exacerbating factors which using one intervention alone cannot prevent the elderly falls [35]. Multifaceted strategies can be very successful, such as training in how to use the toilet, providing safe furniture, securing the home environment, recommendations, and behavioral training which aimed to control and prevent elderly falls due to several factors [36, 37]. Interventions may be effective to reduce falls in the elderly, but if they have extremely high costs, they may not be a viable option. Therefore, policymakers and decision-makers should evaluate both an effectiveness and cost of interventions. Because of the importance of this issue, multifactorial intervention studies can guide policymakers and decision-makers to prioritize and use a combination of these interventions to prevent falls in the elderly [38, 39]. It is necessary to have accurate, clear, and coherent information on the economic costs and cost-effectiveness of these interventions. Therefore, the present study purposes is to review the cost-effectiveness studies of multifactorial interventions and to prevent falls in the elderly through systematic review.

## Methods

The present study is a systematic review conducted in accordance with the Preferred Reporting Items for Systematic Reviews and Meta-Analyses (PRISMA) statement [40, 41]. 

Information Sources and Search Strategy

The required data collected were reviewed between 2000 to February 2019 through the databases of “MEDLINE/PubMed”, Web of Science, Embase, Scopus, Cochrane Library and Google Scholar search engines. The keywords were used includes “Fall injuries”, “Hip fracture”, “Accidental falls”, “Fall”, “Multifactorial interventions”, “Multiple interventions”, “Multifactorial Program”, “Cost-Effectiveness Analysis”, “Cost-Effectiveness”, “Economic Evaluation”, “Cost-Utility Analysis”, “Cost-benefit analysis”, “aged”, “aging”, “elderly”, “older adults”, “older”, “Geriatric” and “Prevention”. Specialized journals and the references of included papers searched manually. The databases of the European Association for Gray Literature Exploitation (EAGLE) and the Health Care Management Information Consortium (HMIC) were also searched (Table 1). Also, Search strategy was developed for other databases according to the databases conditions.

**Table 1:** Complete search strategy for PubMed databases

**Results**	**Strategy**	**Set**
117240	(((“Fall injuries”[Title/Abstract]) OR “Accidental falls”[Title/Abstract]) OR Fall[Title/Abstract]) OR “Hip fracture”[Title/Abstract]	#1
1119	((“Multifactorial interventions”[Title/Abstract]) OR “Multifactorial Program”[Title/Abstract]) OR “Multiple interventions”[Title/Abstract]	#2
69461	((((((“Economic Evaluation”[Title/Abstract]) OR “Cost Benefit Analysis”[Title/Abstract]) OR “Cost Effectiveness”[Title/Abstract]) OR “Cost Effectiveness Analysis”[Title/Abstract]) OR “Cost Utility”[Title/Abstract]) OR “Cost Utility Analysis”[Title/Abstract]) OR “Cost Benefit”[Title/Abstract]	#3
1109204	(((((“aged”[Title/Abstract]) OR “Geriatric”[Title/Abstract]) OR “older”[Title/Abstract]) OR “older adults”[Title/Abstract]) OR “elderly”[Title/Abstract]) OR “aging”[Title/Abstract]	#4
523639	Prevention [Title/Abstract]	#5
3*	#1 AND #2 AND #3 AND #4 AND #5	#6

*Filters activated: Publication date from 2000/01/01 to 2019/02/31.

Eligibility Criteria

All observational studies such as descriptive-analytical, cross-sectional, case-control and cohort were included in the study. Papers presented at conferences and congresses were excluded. Also, studies that did not have adequate information were also excluded via an agreement between the authors.

Review Process

Initially, the titles of founded studies were reviewed and articles that were inconsistent with the study objectives were excluded. Subsequently, abstracts and full texts of the papers were studied and studies that did not meet the inclusion criteria or did not report adequate and appropriate information were identified and excluded. Data were extracted according to the researcher-made data extraction form and entered into the designed form. Endnote X8 was used to organize, study titles and abstracts as well as identify duplicates. The entire review process was conducted by two authors, and disputes were referred to a third person.

Quality Assessment

All included studies were evaluated by two evaluators through the Dramund Economic Studies Quality Assessment Checklist [42]. The Drummond Economic Studies Quality Assessment Checklist has 12 questions. The answers to the checklist’s questions were “Yes”, “No” and “Can’t tell”. “Yes” score 1 and the “No” and “Can’t tell” scored zero. Based on this tool, scores ranged from 1 to 4 rated as poor, between 5 to 8 moderate and 9 to 12 rated as high quality. After the two evaluators review, poor quality papers were excluded and the differences between the two evaluators were referred to as the third evaluator.

Data Extraction and Synthesizing

Content analysis was used to analyze the data, which is a method for identifying, analyzing, and reporting patterns within the text and is widely used in qualitative data analysis [43, 44]. Data were coded by two researchers. The steps for analyzing and coding the data were included familiarity with the text, identifying and extracting primary codes (identifying and extracting more data related to primary codes), identifying themes (inserting extracted primary codes into related themes), reviewing and completing identified themes, naming and defining themes, ensuring the reliability of the extracted codes and themes (agreeing between the two coders through discussion and resolving disputes). The third researcher was referred for consensus in case of disagreement between the two researchers.

## Results

**Fig. 1:**
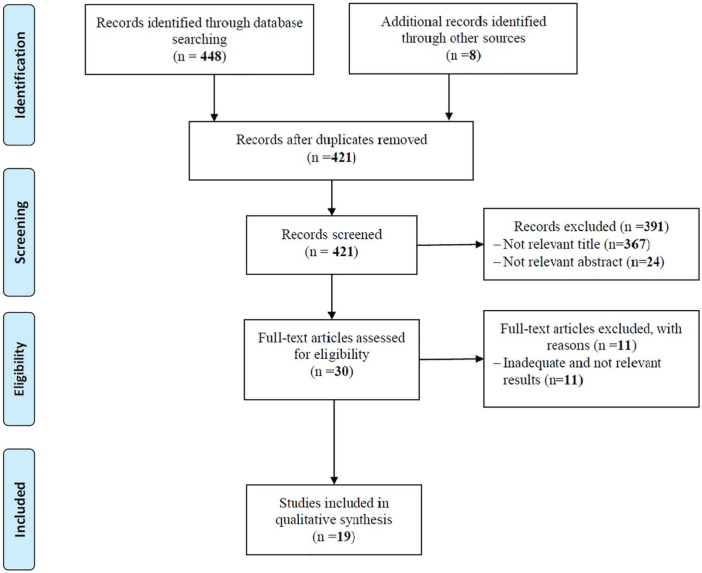
Flow diagram of the searches and inclusion process

Of the 456 studies found in databases search and other information sources, 35 were excluded through screening of the duplicate articles and also, 391 were excluded through the screening of title and abstract. Also, 11 articles were excluded from studying full text due to lack of enough and proper information. Finally, 19 studies were included in the study (Figure 1).

The details and results of the reviewed studies are summarized in Appendix file. Also, the results of the article’s quality appraisal showed that 15 studies were in the high-quality range [39, 45-58] and 4 studies were in the moderate range [38, 59-61] (Appendix file**)**.

Study Results by Studies’ Country

The results of 19 articles found that 16 studies were conducted in 9 countries (USA, Singapore, Australia, Canada, England, the Netherlands, Germany, Finland, and New Zealand) and 3 are multinational studies. The results also showed that 18 out of 19 studies were in high-income countries and only one in Singapore, which is a middle-income country.

Study Results by Place of Studies

The results showed that 16 studies were done at home, two were at the nursing home and one was a Model represent population level. 

Study Results by Studies’ View

In terms of studies’ view, 47% of studies was from a social perspective, 29% from the Health System perspective, and finally 24% from the payer perspective, respectively, which evaluated the cost-effectiveness of multifactorial interventions to prevent falls in the elderly.

Study Results by Type of Interventions

**Fig. 2:**
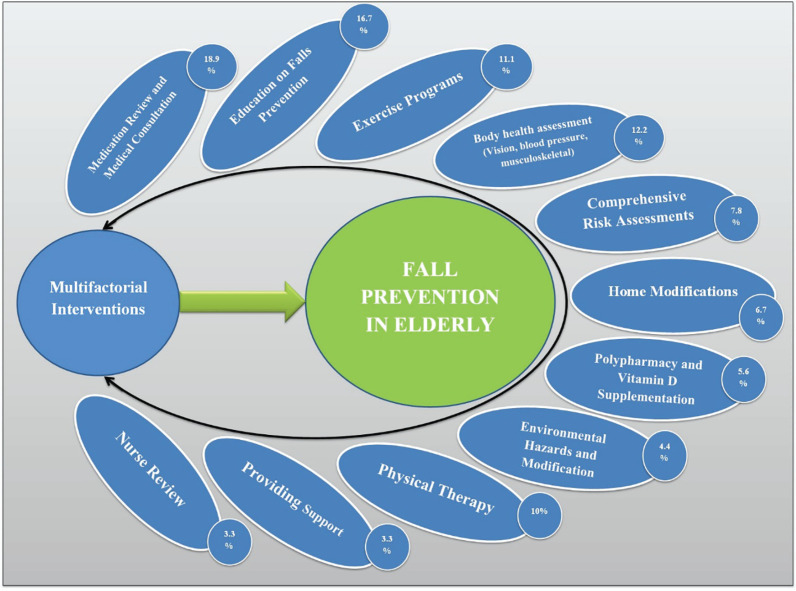
The number of multifactorial interventions to prevent falls in the elderly

The results showed that 72 interventions were performed in 19 studies. The most common interventions were elderly falls in the prevention and medical visits and consultation was 18.9% and elderly education 16.7%, respectively, that the least interventions were nursing care and support services was 3.3% (Figure 2).

Study Results by Type of Economic Evaluation

Fourteen studies were conducted to evaluate the cost-effectiveness of multifaceted interventions and preventing falls in the elderly. Two studies were conducted to evaluate the cost-utility and 3 studies evaluated the Cost-Effectiveness Analysis (CEA), Cost-Utility Analysis (CUA), and Cost-Benefit Analysis (CBA) simultaneously.

Study Results by Included Studies’ Design

From 19 studies in 11 studies, Randomized Control Trial (RCT) with 3320 samples was used to measure effectiveness, and in 3 studies, systematic review of RCT was used. Finally, of the remaining 5 studies, 4 were modeling, and one was RCT simulation, which a total sample size of 11.4 million approximately had been examined. The results showed that 11 studies used secondary data and the rest used primary data.

Study Results by the Amount or Number of Fall Prevention in the Elderly

According to the results, only 9 studies reported the amount or number of fall prevention. A modeling study in the US by Shinyi *et al*. showed that multifactorial interventions reduced the rate of falls in the elderly from 5.697,000 to 513,000 which indicate that 5.184,000 fall has been prevented in the elderly [45]. Also, a 2015 study by Church *et al*. [46] in Australia found that using both systematic review and RCT have reduced about 0.858 by multifactorial interventions elderly falls. Another study conducted by Campbell *et al*. in New Zealand showed that using RCT on 391 samples multifactorial interventions were able to reduce 41% of falls in the elderly as well as prevent 99 cases of falls in the elderly within a year [62]. Also in other studies such as Salkeld *et al*., [51] 14%, the study of GMEE *et al*. [50] 4% and the study of Jenkyn *et al*., in 2012, was 1.29% in the intervention group and 1.37% in the usual-care group which was a multi-country study falls (a difference of 0.08 fewer falls in the intervention group). [49]. These studies had succeeded to reduce falls in the elderly via multifactorial interventions.

Study Results by Life-years Gained and Discount Rate

Of the 19 studies, only 3 studies used a 5% discount rate and one study used a 3% discount rate to calculate the cost-effectiveness of multifactorial interventions [46, 51, 52, 54, 56]. No studies reported information on the life-years gained.

Study Results by Total Cost Reported in Included Studies

All studies except the two were reported the total costs spent on multifactorial interventions. The results of the Wu *et al*., [45] study showed that the total cost of the multifaceted intervention program was US $ 1,879,000,000 for 11.39 million people, which reduced the costs of elderly falls from the US $ 32,088,000,000 to the US $ 2,674,000 in one year. Also in a study of Matchare *et al*., [61] that used the RCT method, the total cost was S $ 3718 for the intervention group and S $ 3356 for the control group for each participant in 9 months multifactorial interventions. Study of Church *et al*., [54] showed that the average cost for multifactorial interventions is AUD 4991. In this study, multifactorial interventions were considered as the most expensive interventions to prevent elderly falls. Another study [49] reported that the average cost of multifactorial interventions was an average of $ 18,916 in the intervention group and $ 8,973 in the usual-care group. The results showed that the cost for multifactorial interventions as an effective strategy, fluctuated from $ 272 per person as the lowest cost in the study of Frick *et al*., [60] in 2010 to £ 880 per person as the highest costs in the 2010 by Colleen *et al*., [53].

Study Results by Cost Per QALY Reported in Included Studies

Of the 19 studies, 7 reported gained QALY as a result of multifactorial interventions. According to the results of a study by Matchare *et al*., [61]in Singapore, the amount of gained QALY by multifactorial interventions was 0.003 per person. Also the results of Church *et al*., [46] study showed that 1.276 QALYs were gained through multifaceted interventions. Another study in the Netherlands showed that the mean QALY in the intervention group as a result of multifactorial interventions was 0.76 per person [50]. Study of Church *et al*., [54] study showed that the implementation of multifactorial interventions QALY gained was $ 125.868 in intervention with only referral and $ 165,841 in the active component. Also, the results of the study showed that the cost of each gained QALY at Multifactorial-active was $ 130139 and in Multifactorial-referral was $ 172009. The results of a study conducted by Muller *et al*., [56] in 2015 showed that gained QALY per person is 1.226 and the study of Farag *et al*., [55] in Australia showed that the cost of each QALY obtained through multifactorial interventions was $ 28,931.

Study Results by Cost-effectiveness ICER in Included Studies

Of the 19 studies, 14 were reported cost-effective ICER of multifactorial interventions. The study results of Wu *et al*., [45] showed that the cost-effectiveness ratio per person to prevent elderly falls was less than $ 1500. Also, the results of the Matchare *et al*., [61]study in 2018 showed that ICER was the US $ 120,667 for a multifactorial intervention program to prevent elderly falls in Singapore. The results of another study conducted by Heinrich et al., [39] in Germany, 2013 showed that the ICER was EUR 7,481 per year for multifactorial interventions. Another study which conducted in Germany showed that ICER was € 21,353 for each QALY [56]. The ICER for multifactorial interventions for each prevented elderly fall ranged from the US $ 120,667 [39] to the US $ 4280.9 [57].

Study Results by the Overall Results Obtained from Multifactorial Interventions 

The results showed that most studies do not consider multifactorial interventions as cost-effective as the usual care or single-factorial interventions provided for the elderly to prevent and treat the traumatic injury. The results of large-sample modeling studies showed that multifactorial interventions are cost-effective and reduce the costs of elderly falls in the future.

## Discussion

Of the 456 studies, 19 were finally included in the study. Most studies (18 studies) were in high-income countries. Most studies (16 studies) were done at the community level. The medical visit and consultation and elderly education were the most common interventions. Most of the studies have done with the purpose of cost-effectiveness and using the RCT method. The preventing cost of each fall ranged from $ 272 to $ 880 per person. Also the range of multifactorial interventions ICER rang was from the US $ 120,667 to the US $ 4280.9.

The results showed that most studies were done in high-income countries. One reason for this could be the high prevalence of aging in high-income countries [63, 64]. Other reasons could be the availability of research infrastructures and the economic potential of these countries to implement multifactorial interventions because the design and implementation of these interventions require a large and powerful executive and research team to prevent elderly falls and it costs a lot. High-income countries face fewer problems to design and implement multifaceted interventions because of their high economic capacity and the allocation of sufficient funds for research, especially in the field of aging. However, in low- and middle-income countries, the mortality of fall-related injuries in the elderly and the high costs of the mortality, aging, and prevention of elderly falls has become very important for these countries because of the epidemiological transition and the growing trend of the elderly population [65, 66]. To implement interventions in regards of preventing elderly falls, we should pay attention to experiences of high-income countries and it can be a good guide for modeling, planning and implementing of these intervention according to the specific conditions of the low-income countries.

The results of the present study showed that most studies were done at Home level, therefore, it can be because of the length of elderly presence at this level and most of the elderly falls occur in this location. Studies in this area also show that one-third of the elderly who were presented in the community fall at least once a year, while, this is more likely for those elderly who are physically weaker [67-70]. Other possible reasons for further home level studies includes high a control ability variable and confounders in the home environment compared to other areas are low awareness of family members comparing with a nursing home and other centers providers, and also supervision lack to adhere to the principles of the home safety. 

In general, interventions of elderly fall prevention can be classified into two parts: single-factorial and multifactorial interventions [71-74]. Theoretically, multifactorial interventions are more effective in preventing elderly falls, but studies and documentation show that multifactorial interventions are less cost-effective than other types of single-factor interventions [46, 48-50, 52, 55, 75, 76]. It should be noted that multifactorial interventions had acceptable effectiveness, but their cost-effectiveness decreased because of their design high costs and implementation. To increase the cost-effectiveness of multifactorial interventions, for example, interventions such as elderly and their families education about safety tips in-home and community, combining it with medical visits and consultation both are cost-effective and efficient to reduce elderly fall. 

The results of the study showed that 19 multifactorial interventions that evaluated were composed about 72 single-factor interventions. The most common were physician visit and medical consultation (18.9% of total interventions) [45, 48, 49], elderly education (16.7% of the total interventions) [38, 56, 58, 61], exercise programs (11.1% of all interventions) [45, 50, 53], and evaluation of the elderly’s physical health (level of vision, blood pressure, and muscle strength) (12.2% of the total interventions) [46, 53], and the least common interventions includes nursing [47] and supportive care [38]. The greater interventions’ use was medical consultation and elderly education that can be low cost and easy implementation at a broader level. Whereas, interventions such as Occupational-therapy, home safety assessment, and modification require more time and cost because of the make changes need to the community and home environment and reduce environmental hazards. Therefore, it is recommended focusing on interventions that are less easily implemented and less costly, and considering the effectiveness of single-factorial interventions in different studies and an appropriate use combination of them to design and implement multifaceted interventions.

Medical visit and consultation is an effective intervention that conducted in a team setting of multifactorial interventions (consultation by a pharmacist, physician, and treatment team), and as one of the multifactorial interventions components which has been used repeatedly. For example, using of pharmacological consultation and the removal of psychotropic drugs in the elderly can significantly reduce the rate of elderly falls based on various studies [77, 78]. Medical visits and consultation is a cost-effective both as a single-factorial intervention [77, 78] and as part of multifactorial interventions for reasons such as implementation ease, low cost, broad applicability, and its high effectiveness in preventing elderly falls compared to other interventions [46]. 

Elderly education in preventing falls is another intervention that has been used more frequently than other interventions [38, 56, 58, 61]. The reasons for repeated use of this type of intervention can be due to its complementarity nature with other multifaceted interventions, its low cost and ease of implementation as well as the coverage of a large population of the elderly, which can have a great impact on increasing the effectiveness of multifactorial interventions. Although, some evidence suggests that the effectiveness of education in prevention of elderly falls is low [79], other evidence indicates that this method can be used as an effective and cost-effective method if appropriate education methods used rationally and appropriately [80-82].

The results of the study related to the type of economic evaluation showed that the most of studies exanimated cost-effectiveness. Only two studies examined the cost-benefit of the multifactorial interventions, Kevin *et al*., [60] in 2010 showed that multifactorial interventions were costly and ineffective compared to single-factor interventions, whereas in a 2015 study by Dirk *et al*., [56] multifactorial interventions were described as a cost-effective interventions to prevent multiple fractures in nursing homes.

The results showed that the most studies done by the RCT method and the rest of the studies were modeling and systematic review. Modeling studies have evaluated the cost-effectiveness of multifactorial interventions with a very large sample size and the most of these studies have confirmed multifactorial interventions as a cost-effective method [39, 45, 56, 60]. This could be due to modeling with very large sample size and at a large area, which significantly reduces elderly falls and shows the cost of interventions to prevent elderly falls and the imposed economic burden very low. However, the most RCT studies did not confirm the cost-effectiveness of multifactorial interventions to prevent elderly falls [38, 47-51, 54]. One of the most important reasons for the results of RCT studies is the high accuracy of these studies in data controlling and the proximity of these studies to reality, since the studies are implemented in the community with considering all aspects. Therefore, the results of RCT studies can be more reliable and realistic. Modeling or simulation studies are less reliable, despite being conducted at a large community level, due to poor community control and lack of real data.

One of the important effects of multifactorial interventions, is the prevention of elderly falls and gained life years. Nevertheless, none of the included studies took into account the gained life years. Therefore, it is better to consider and calculate the gained life years in other studies in this field. Also, the amount or number of preventable falls in the elderly were reported in only 9 studies, which requires a great deal of attention in economic evaluation studies. Wu *et al*., [45] has studied 11.39 million elderly people in Medicare and Medicaid which using population-based modeling, and indicate that multifactorial interventions can reduce elderly falls from 5.697,000 to 513,000 in one year. In RCTs studies, evaluating multifactorial interventions that affect elderly falls, were reported multifactorial interventions more costly in comparing with single-factorial interventions and non-intervention. 

The overall results of the included studies showed that although multifactorial interventions are effective and reduce the rate of elderly falls, economic evaluation of these interventions through ICER, aims to economically evaluate intervention policy or not intervention, demonstrated that multifactorial interventions do not cost-effective.

Multifactorial interventions can reduce effective factors of elderly falls by considering many benefits such as evaluating and identifying different aspects of risk factors, gaining support and collaboration of participants in intervention, and combining different and complementary interventions to increase the effectiveness of interventions. Also, the results of various studies and guidelines of the American and British Surgeons’ Association have suggested multifactorial interventions as one of the most effective interventions and one of the main strategies in preventing elderly falls [83-85]. From the limitations of designing multifactorial interventions to prevent elderly falls, it is understood that they are just a combination of single-factorial interventions and multi-factorial interventions which planned and targeted and another single factor intervention adds to it only if covers a weak dimension of multifactorial intervention. Another limitation of multifactorial interventions is the time-consuming and costly design of these interventions. Also, since multifactorial interventions are designed from a combination of single-factorial interventions, the effects evaluating of these interventions separately is difficult and ambiguous, therefore, the effects of the combined single-factorial interventions cannot be evaluated separately and determine which factor has been weak or very effective. Due to the difference between multifactorial and single-factorial interventions, multi-factorial interventions can be more difficult and costly because of the combination of multiple factors. Also, it can cause confusion in elderly and reduce the effectiveness of multifactorial intervention components [62, 86].

**Limitations:** One of the limitations of this study is that the research team were used only the English language to search for data. This was also due to the authors’ lack of skill in other languages and to the data type that obtained in this study. Therefore, it was not possible to perform meta-analysis in this study.

**Strengths:** On the other hand, aging is on the rise in all societies and will the risk of aging falls. Given this, countries need to design and implement effective interventions in this area. One of the most effective interventions to prevent the fall of the elderly is multifactorial interventions. One of the strengths of the present study is that the results of this study can help policymakers in this area for evidence-based decision-making by providing appropriate and comprehensive information.

## Conclusion

According to the results, multifactorial interventions can be considered as one of the most effective strategies to prevent elderly falls. Most high-income countries implement these interventions because of the high cost of interventions’ designing and implementing on a large scale. Despite their high effectiveness, these interventions are not cost-effective because of their high cost. To design and implement multifactorial interventions, it is recommended to carefully select and combine single-factorial interventions that are resource-efficient, low cost, a good complement to other interventions, and more adaptable to country-specific conditions.

## Funding sources:

This study was supported by Health Management and Economics Research Center, Iran University of Medical Sciences (IUMS) (Grant number: IR.IUMS.REC.1397.1344). 
